# Antibody-Functionalized MoS_2_ Nanosheets for Targeted Photothermal Therapy of *Staphylococcus aureus* Focal Infection

**DOI:** 10.3389/fbioe.2019.00218

**Published:** 2019-09-10

**Authors:** Yuqian Zhang, Weijun Xiu, Siyu Gan, Jingyang Shan, Shaokang Ren, Lihui Yuwen, Lixing Weng, Zhaogang Teng, Lianhui Wang

**Affiliations:** ^1^Key Laboratory for Organic Electronics and Information Displays & Jiangsu Key Laboratory for Biosensors, Jiangsu National Synergetic Innovation Centre for Advanced Materials (SICAM), Institute of Advanced Materials (IAM), Nanjing University of Posts and Telecommunications, Nanjing, China; ^2^Laboratory of Immunology and Nanomedicine, Guangdong Key Laboratory of Nanomedicine, Institute of Biomedicine and Biotechnology, Shenzhen Institutes of Advanced Technology (SIAT), Chinese Academy of Sciences, Shenzhen, China; ^3^School of Geography and Biological Information, Nanjing University of Posts and Telecommunications, Nanjing, China; ^4^Department of Medical Imaging, School of Medicine, Jinling Hospital, Nanjing University, Nanjing, China

**Keywords:** targeted photothermal therapy, *Staphylococcus aureus*, infection, antibody, MoS_2_ nanosheets

## Abstract

Bacterial biofilm-related diseases cause serious hazard to public health and bring great challenge to the traditional antibiotic treatment. Photothermal therapy (PTT) has been recognized as a promising alternative solution. However, the therapeutic efficacy of PTT is often compromised by the collateral damage to normal tissues due to the lack of bacteria-targeting capability. Here, a *Staphylococcus aureus* (*S. aureus*)-targeted PTT nanoagent is prepared based on antibody (anti-protein A IgG), polydopamine (PDA), and PEG-SH (thiolated poly (ethylene glycol)) functionalized MoS_2_ nanosheets (MoS_2_@PDA-PEG/IgG NSs, MPPI NSs). The PDA was used as bio-nano interface to facilitate the covalent conjugation of antibody and PEG-SH onto the surface of MoS_2_ NSs via facile catechol chemistry. Targeted PTT of MPPI NSs shows excellent inactivation efficiency of larger than 4 log (>99.99%) to *S. aureus* both in biofilms (*in vitro*) and in infected tissues (*in vivo*) without causing damage to normal mammalian cells. By contrast, non-targeted PTT of MoS_2_@PDA-PEG NSs (MPP NSs) only kills *S. aureus* by <90% *in vitro* and <50% *in vivo*. As a result, *S. aureus* focal infection in mice healed much faster after PTT of MPPI NSs than that of MPP NSs. The superiority of targeted PTT may originate from the efficient accumulation and close binding of PTT agents to bacterial cells. Therefore, MPPI NSs with bacteria-targeting capability are promising photothermal agents for effective treatment of *S. aureus* focal infection.

## Introduction

*S. aureus* is one of the major causes for infectious diseases, such as skin and sinus infections, endocarditis, bacteraemia, and so on, and brings severe threats to human health (van Hal et al., 2012; Wolcott et al., 2016; Hasan et al., 2017). Most of these infectious diseases are relevant to the formation of bacterial biofilms, which are organized aggregates of bacterial cells encased in extracellular polymeric substances (EPSs) on natural or abiotic surface (Costerton et al., 1999; Davies, 2003). The bacteria in biofilms develop much higher resistance to antibiotics and the host defense system than planktonic bacterium, making it a great challenge for conventional chemotherapy to treat bacterial biofilm-related infections (Costerton et al., 1995; Lynch and Robertson, 2008; Penesyan et al., 2015). Other therapeutic methods, such as surgical remove combined with long-term antibiotic therapy, would augment patients' suffering and incur high healthcare costs (Duncan et al., 2015). Therefore, an urgent need of alternative antibiofilm strategies arises.

PTT is a simple, efficient, and non-invasive method to kill bacteria by using near-infrared (NIR) light induced local hyperthermia (Ray et al., 2012). Since bacteria in biofilms have little capability to resist the heat or stop the heat transfer, PTT is less possible to evoke bacterial resistance than antibiotics (Yuwen et al., 2018). Thus, PTT possesses great potential for the treatment of bacterial biofilm-related infections. Many nanomaterials have been used as PTT agents for the photothermal destruction of bacterial biofilms, such as various gold-based nanostructures and carbon nanomaterials (Jo and Kim, 2013; Levi-Polyachenko et al., 2014; Pallavicini et al., 2014; Ji et al., 2016; Meeker et al., 2016, 2018; Teng et al., 2016; Hu et al., 2017). However, relatively high temperature is usually needed to eliminate bacterial biofilms completely by PTT, which would cause harm to surrounding healthy tissues and limit the application of PTT (Hauck et al., 2008; Hsiao et al., 2015). In order to solve this problem, it is necessary to improve the accumulation of photothermal agents in bacterial biofilms and reduce the distance between photothermal agents and bacterial cells. The integration of bacteria-targeting moieties and photothermal agents would be a possible solution.

MoS_2_ nanosheets (MoS_2_ NSs) are promising photothermal agents due to their large surface area, good biocompatibility, high extinction coefficient, and high photothermal conversion efficiency in the NIR region (Robinson et al., 2011; Chou et al., 2013; Li et al., 2017). Zhang et al. (2016) have prepared chitosan functionalized MoS_2_ to combat bacterial infection by NIR-triggered sterilization. Yin et al. (2016) combine the peroxidase-like activity and PTT ability of MoS_2_ nanoflowers, realizing a rapid and effective killing of bacteria *in vitro* and wound disinfection *in vivo*. Yuwen et al. (2018) prepared MoS_2_ NSs-silver nanoparticles composites to improve the antibiofilm efficacy via photothermal enhanced release of silver ions. Although MoS_2_ NSs-based PTT has proven effective to treat bacterial infections, their further application is still hindered due to the lack of bacteria-targeting capability.

**Scheme 1:**
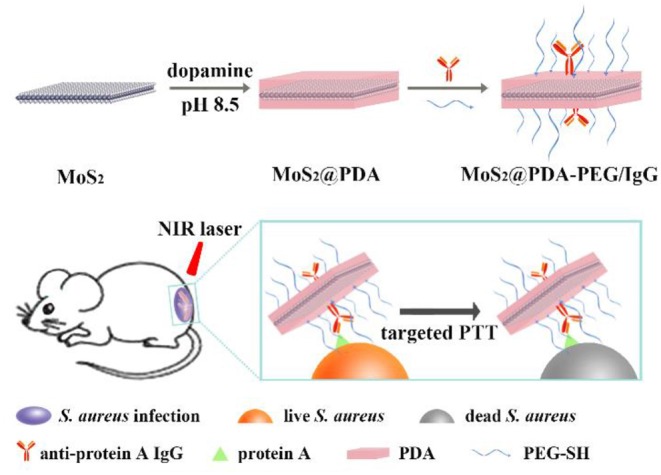
The preparation of MoS_2_@PDA-PEG/IgG NSs (MPPI NSs) and their application for targeted PTT of *S. aureus* focal infection.

Here, MoS_2_@PDA-PEG/IgG NSs (MPPI NSs) with *S. aureus*-targeting capability and photothermal properties were prepared by coating MoS_2_ NSs with polydopamine (PDA) and subsequently conjugating of anti-protein A IgG and PEG-SH (Scheme 1). With good biocompatibility, colloidal stability, and high photothermal effect, MPPI NSs were used for the treatment of *S. aureus* biofilms *in vitro* and *S. aureus* focal infection *in vivo* successfully with much higher therapeutic efficacy than non-targeted PTT.

## Materials and Methods

### Materials

Molybdenum disulphide (MoS_2_) powder (<2 μm, 99%) and bovine serum albumin (BSA) were purchased from Sigma-Aldrich, and n-butyllithium (n-BuLi, 2.4 M hexane solution) from Amethyst. Dopamine hydrochloride (99%) was obtained from Alfa Aesar. Thiolated poly (ethylene glycol) methyl ether (PEG-SH, M_w_ = 5000) and rabbit anti-protein A IgG (IgG) were bought from JenKem Technology and Beijing Biosynthesis Biotechnology Co., LTD, respectively. Ultrapure water (Millipore, 18.2 MΩ) was used to prepare aqueous solutions throughout the whole study.

### Preparation of MoS_2_ NSs

The ultrasonication enhanced lithium intercalation (ULI) method we reported previously was used to prepare single-layer MoS_2_ NSs (Zhang et al., 2017).

### Preparation of MoS_2_@PDA NSs (MP NSs)

Tris-HCl buffer (10 mM, pH = 8.5, 20 mL), MoS_2_ NSs aqueous dispersion (1 mg/mL, 0.5 mL), and dopamine hydrochloride aqueous solution (10 mg/mL, 0.3 mL) were added into a 50 mL microwave reaction tube, and reacted in the microwave reactor (Explorer 48, CEM) at 60°C for 10 min (Yuwen et al., 2018). The reaction mixture was centrifuged at 12,000 rpm for 20 min. And the sediment was resuspended in ultrapure water. After repeating the centrifugation twice, MP NSs were resuspended in ultrapure water.

### Preparation of MoS_2_@PDA-PEG/IgG NSs (MPPI NSs)

Tris-HCl buffer (10 mM, pH = 8.5, 5 mL), rabbit anti-protein A IgG aqueous solution (IgG, 1 mg/mL, 1 mL), and PEG-SH aqueous solution (50 mg/mL, 0.33 mL) were added into a 50 mL centrifuge tube and mixed evenly. MP NSs aqueous dispersion (MoS_2_: 1 mg/mL, 1 mL) was added. The reaction mixture was incubated overnight in an orbital shaker at 220 rpm and 37°C. After centrifugation (12,000 rpm, 20 min) for three times, MPPI NSs were resuspended in ultrapure water.

### Preparation of MoS_2_@PDA-PEG NSs (MPP NSs)

The MPP NSs were prepared using the similar method with the preparation of MPPI NSs except the addition of IgG.

### Quantitative Analysis of Protein Loading

Due to the cost reason, frequently-used protein bovine serum albumin (BSA) was used instead of IgG to evaluate the protein loading efficiency of MP NSs and MoS_2_ NSs by using Bradford protein assay (Compton and Jones, 1985). Bradford reagent was prepared by dissolving Coomassie Brilliant Blue G-250 (10 mg) into ethanol solution (95%, 5 mL), and then adding phosphoric acid solution (85%, 10 mL) and H_2_O (85 mL).

Tris-HCl buffer (10 mM, pH = 8.5, 5 mL), BSA aqueous solution (1 mg/mL, 1 mL), PEG-SH aqueous solution (50 mg/mL, 0.33 mL), and MoS_2_ NSs or MP NSs aqueous dispersion (MoS_2_: 1 mg/mL, 1 mL) were mixed evenly in 50 mL centrifuge tubes, respectively. The reaction mixtures were incubated overnight in an orbital shaker at 220 rpm and 37°C. After centrifugation (12,000 rpm, 20 min) for five times, the supernatant was collected.

Supernatant (50 μL) and BSA aqueous solutions at different concentrations (8, 16, 32, and 64 μg/mL, 50 μL) were mixed with Bradford reagent (250 μL), respectively. After 5 min, the absorbance at 595 nm of these mixtures was measured. A working curve of absorbance at 595 nm vs. concentration of BSA was built as followed, C (μg/mL) = (OD_595_—0.072)/0.00456. The amount of BSA in the supernatant was determined by using the above equation. Finally, the amount of protein integrated to MoS_2_ NSs and MP NSs was calculated according to the total amount of BSA added.

### Cytotoxicity Assay

Minimum essential medium (MEM, KeyGEN BioTECH, containing penicillin-streptomycin) with supplement of fetal bovine serum (FBS, Gibco, 10%) and trypsin-EDTA (0.25% w/v) were used to culture and detach the human cervical carcinoma (HeLa) cells (KeyGEN BioTECH), respectively. MoS_2_ NSs, MP NSs, MPP NSs, and MPPI NSs suspended in MEM (no FBS, 200 μL/well) were added into HeLa cells that grew in 96-well plates. After incubation for 24 h, the lactate dehydrogenase (LDH) from the supernatant was detected using the LDH-cytotoxicity colorimetric assay kit (BioVision) and the cytotoxicity of nanosheets was evaluated following the instruction described in our previous work (Zhang et al., 2017). A microtiter plate reader (PowerWave XS2, BioTek) was used to measure the optical density at 495 nm (OD495).

### Photothermal Toxicity of MPPI NSs

The PTT of MPPI NSs was carried out on the human prostatic stromal myofibroblast cell line WPMY-1 cells to evaluate the side effects. The WPMY-1 cells were seeded in 96-well plates using DMEM (Dulbecco's modified Eagle's medium, 10% FBS, 80 U/mL penicillin, and 0.08 mg/mL streptomycin). After 24 h, WPMY-1 cells were washed with sterile saline, and incubated with different concentrations of MPPI NSs (containing 0, 40, 80, and 160 μg/mL MoS_2_) suspended in DMEM (no FBS) for another 6 h. The cells were rinsed with saline, and 50 μL of DMEM (no FBS) was added into each well. The NIR laser irradiation was performed using a 785 nm laser at 0.58 W/cm^2^ for 10 min. The supernatants were transferred to another 96-well plate to calculate the cell viability using the LDH-cytotoxicity colorimetric assay kit as we described above. Adherent cells were co-stained by calcein-AM and propidium iodide (PI, KeyGEN BioTECH), and imaged using an Olympus IX71 inverted fluorescence microscope.

### Bacteria Culture and Biofilm Formation

*S. aureus* ATCC 25923 and *Pseudomonas aeruginosa* PA01 were maintained on Luria-Bertani (LB) agar and stored at 4°C. Before use, a single isolated colony was transferred into LB broth in a 50 mL centrifuge tube and incubated at 37°C in a rotary incubator with shaking at 200 rpm overnight. After rinsed with sterile saline twice, the bacteria were suspended in fresh LB medium supplemented with 1% glucose (LBG broth) at a concentration of 10^7^ colony forming units (CFU)/mL. The bacterial suspension was added into 6-well plates with ITO glass with a volume of 3 mL/well, 96-well plates with a volume of 200 μL/well, and glass-bottomed culture dishes (confocal dishes) with a volume of 3 mL/well, respectively. Established biofilms were obtained after incubation at 37°C for 24 h (Chen et al., 2016).

### *In vitro* PTT of *S. aureus* Biofilms

*S. aureus* biofilms grown in 96-well plates were rinsed softly and cultured with MPPI NSs and MPP NSs (MoS_2_: 0, 40, 80, and 160 μg/mL) suspended in saline at 37°C, respectively. After 6 h, unbound nanosheets were washed away. Sterile saline (50 μL) was added to maintain the humidification of biofilms. NIR laser irradiation was performed by using a 785 nm continuous-wave laser at the power density of 0.58 W/cm^2^ for 10 min. These biofilms treated with both nanosheets incubation and NIR laser irradiation were classified into MPP + NIR and MPPI + NIR groups. The biofilms with only nanosheets incubation and only NIR laser irradiation (MoS_2_: 0 μg/mL) were set as control.

The bacteria inside biofilms of each well were dispersed into saline thoroughly by pipetting. Bacterial viability was determined by serial dilution and plate counting as CFU per well.

### SEM Imaging of *S. aureus* Biofilms

*S. aureus* biofilms grown on ITO glass after 6 h incubation with MPPI NSs or MPP NSs and *P. aeruginosa* biofilms grown ITO glass after 6 h incubation with MPPI NSs were washed with saline twice, fixed in 2.5% glutaraldehyde for 30 min, dehydrated with graded ethanol series (25%, 50%, 75%, and 100%) for 10 min each, sputter-coated with gold, and imaged by SEM.

The morphology change of *S. aureus* biofilms after PTT with MPPI NSs and MPP NSs were also observed by SEM after similar treatments consisting of fixing with glutaraldehyde, dehydrating with graded ethanol series, and sputter coating with gold.

### Quantitative Assessment of Biofilm-Binding by Energy Dispersive Spectroscopy (EDS)

After incubation with MPPI NSs or MPP NSs for 6 h, the binding affinity of MPP NSs and MPPI NSs to *S. aureus* biofilms, and that of MPPI NSs to *P. aeruginosa* biofilms were assessed. Biofilms grown on ITO glass were washed with saline twice after incubation with MPPI NSs and MPP NSs, fixed in 2.5% glutaraldehyde for 30 min, dehydrated with graded ethanol series (25, 50, 75, and 100%) for 10 min each, and examined by EDS.

### Crystal Violet Staining Assay of *S. aureus* Biofilms

Crystal violet staining was used for the structure observation of *S. aureus* biofilms. The biofilms in 96-well plates treated with MPPI NSs incubation (MoS_2_: 0, 40, 80, and 160 μg/mL) and NIR laser irradiation were all fixed with 2.5% glutaraldehyde for 30 min, and stained with 0.2% crystal violet for 1 h. Excessive dye was rinsed by saline. Biofilms were imaged using an inverted microscope (Olympus IX71).

### Three Dimensional (3D) Confocal Laser Scanning Microscopy (CLSM) Observation

*S. aureus* suspended in LBG broth at the concentration of 10^7^ CFU/mL was added into confocal dishes with a volume of 3 mL/well, and cultured at 37°C for 24 h. Then, *S. aureus* biofilms were treated with different conditions: only MPP NSs incubation (MPP), only MPPI NSs incubation (MPPI), MPP NSs incubation and NIR laser irradiation (MPP + NIR), and MPPI NSs incubation and NIR laser irradiation (MPPI + NIR). Biofilms were stained by calcein-AM (KeyGEN BioTECH) for 20 min, washed with sterile saline to remove excessive dye, and imaged by Olympus IX81 confocal laser scanning microscope with an imaging area of 630 × 630 and 1 μm interval on z-section.

### *In vivo* PTT of *S. aureus* Focal Infection

All animal procedures were performed in accordance with the Guidelines for Care and Use of Laboratory Animals of Nanjing University and experiments were approved by the Animal Ethics Committee of Nanjing University. Six-week-old female Balb/c mice (Nanjing Junke Biological Engineering Co. Ltd.) were first anesthetized and depilated. *S. aureus* suspended in LBG broth at the concentration of 10^9^ CFU/mL was injected into the right flanks of mice subcutaneously with a volume of 40 μL each to construct subcutaneous abscesses. After 24 h, 48 mice with *S. aureus* focal infection were randomly divided into six groups and injected *in situ* (into the abscess) with 100 μL saline, 100 μL MPP NSs (suspended in saline, MoS_2_: 40 μg/mL), and 100 μL MPPI NSs (suspended in saline, MoS_2_: 40 μg/mL), respectively. The NIR laser irradiation was performed at 12 h post-injection using a 785 nm laser at the power density of 0.58 W/cm^2^ for 10 min. The sizes of abscesses were measured by using a caliper every other day, and photographs were taken at the same time. All mice were executed at 8th day. The infected tissues of three mice from each group were dissected and fixed in 10% neutral buffered formalin for hematoxylin and eosin (H&E) staining and Masson's trichrome staining. The stained slices were imaged by using an Olympus IX71 microscope. The *S. aureus* infected tissues of five mice from each group were transferred into sterile saline. The bacteria of abscesses were dispersed thoroughly by ultrasonication to determine the CFU by plating.

### *In vivo* Toxicity Assessment of MPP NSs and MPPI NSs

Nine female Balb/c mice (6-week-old) were divided randomly into three groups, and were intravenously (i.v.) injected with saline, MPP NSs, and MPPI NSs suspended in saline (MoS_2_: 500 μg/mL) with a volume of 200 μL, respectively. One month after the injection, these mice were all sacrificed and their major organs (heart, liver, spleen, lung, and kidney) were collected for H&E staining.

## Results

### Morphology and Properties

**Figure 1:**
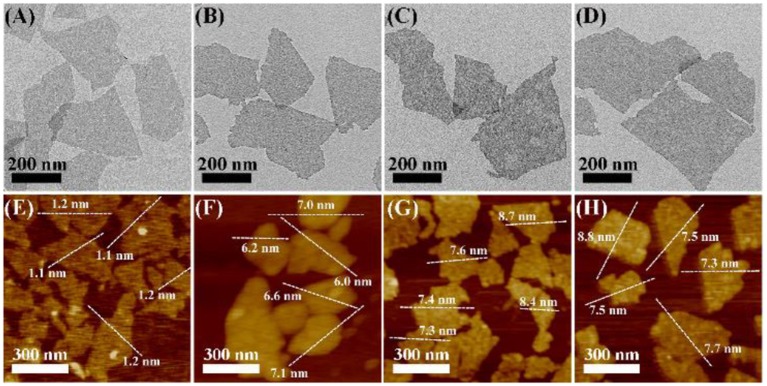
Transmission electron microscopy (TEM) images **(A–D)** and atomic force microscopy (AFM) images **(E–H)** of MoS_2_ NSs, MP NSs (MoS_2_@PDA NSs), MPP NSs (MoS_2_@PDA-PEG NSs), and MPPI NSs (MoS_2_@PDA-PEG/IgG NSs), respectively.

As shown in Figures 1A,E and Figure S1, the transmission electron microscopy (TEM) and atomic force microscopy (AFM) images show that the as-prepared MoS_2_ NSs have uniform single-layer sheet-like morphology with sizes of 100~500 nm and thickness of about 1.2 nm, consistent with previous reports (Yuwen et al., 2016). As shown in Figures 1A–H, MoS_2_@PDA NSs (MP NSs), MoS_2_@PDA-PEG NSs (MPP NSs), and MoS_2_@PDA-PEG-IgG NSs (MPPI NSs) show no obvious change in terms of size and shape compared with MoS_2_ NSs. After PDA coating, the thickness of MP NSs rises to about 6.6 nm (Figure 1F, Figures S1D,F). The thicknesses of MPP NSs and MPPI NSs increase to 7.8~7.9 nm (Figures 1G,H). The hydrodynamic sizes of MoS_2_ NSs, MP NS, MPP NSs, and MPPI NSs determined by dynamic light scattering (DLS) are ~225 nm, ~246 nm, ~271 nm, and ~261 nm, respectively (Figure S2A). As shown in Figure S2B, the zeta potential of MoS_2_ NSs is about −32 mV, and that of MP NSs is about −22 mV. The electroneutral PEG-SH increases the zeta potential of MPP NSs to about −17 mV, and the zeta potential of MPPI NSs is about −27 mV.

**Figure 2:**
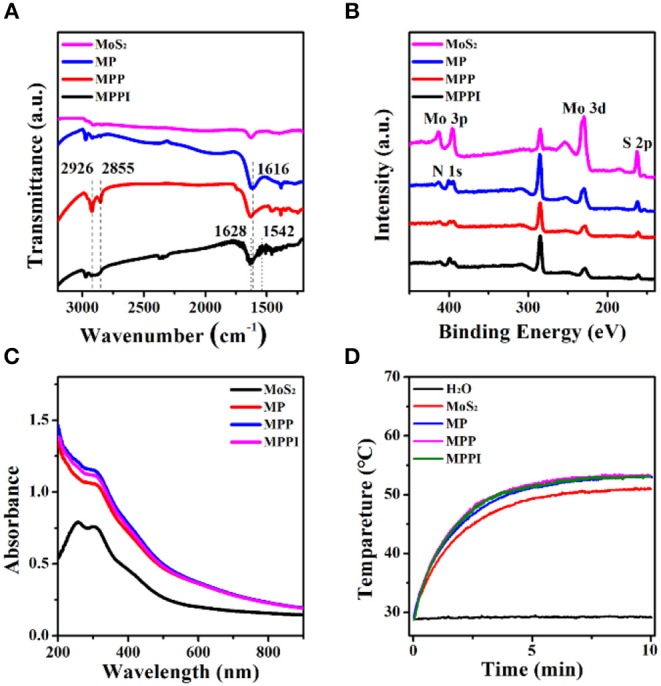
**(A)** Fourier transform infrared (FT-IR) spectra, **(B)** X-ray photoelectron spectroscopy (XPS) spectra, and **(C)** Ultraviolet-visible-near infrared (UV-Vis-NIR) absorption spectra of MoS_2_ NSs, MP NSs, MPP NSs, and MPPI NSs. **(D)** Photothermal heating curves of MoS_2_ NSs, MP NSs, MPP NSs, and MPPI NSs (MoS_2_: 30 μg/mL). All samples were irradiated under 785 nm laser at the power density of 0.43 W/cm^2^ for 10 min.

As shown in Figure 2A, the IR absorption band near 1,616 cm^−1^ of MP NSs can be assigned to the C = C stretching vibration and N-H bending vibration from PDA, suggesting the successful coating of PDA on the surface of MoS_2_ NSs (He et al., 2014). The C-H stretching vibrations at 2,926 and 2,855 cm^−1^ demonstrate the presence of PEG-SH in both MPP NSs and MPPI NSs (Yuan et al., 2015; Uppu et al., 2016). The characteristic peaks of Amide I (1,628 and 1,624 cm^−1^) and Amide II (1,578 and 1,542 cm^−1^) from MPPI NSs proves the conjugation of IgG (Figure 2A and Figure S3; Islam et al., 2017). The X-ray photoelectron spectroscopy (XPS) survey spectra of MoS_2_ NSs, MP NSs, MPP NSs, and MPPI NSs are shown in Figure 2B. The binding energy peaks of Mo 3p_1/2_ (~412 eV), Mo 3p_3/2_ (~394 eV), Mo 3d (~229 eV), and S 2p (~162 eV) can be observed in MoS_2_ NSs (Kibsgaard et al., 2012; Ganta et al., 2014). The intensity of these characteristic peaks of MoS_2_ decrease in MP NSs, MPP NSs, and MPPI NSs after the surface modification, and N 1s (~399 eV) peak exists in all these three nanosheets, demonstrating the presence of PDA and IgG (Ryou et al., 2011).

As shown in Figure 2C, MoS_2_ NSs have broad absorption ranging from the ultraviolet to the NIR region. The absorbance of MP NSs increases slightly due to the PDA coating (Liu et al., 2014). The ultraviolet-visible-near infrared (UV-Vis-NIR) absorption spectra of MPP NSs and MPPI NSs are almost the same as that of MP NSs. Figure 2D shows that the temperature of water increased <1°C under 785 nm laser irradiation at 0.43 W/cm^2^ for 10 min, while the temperature of MoS_2_ NSs aqueous dispersion (30 μg/mL) reached as high as 50°C. Meanwhile, the final temperatures of MP NSs, MPP NSs, and MPPI NSs aqueous dispersions containing 30 μg/mL of MoS_2_ were ~2°C higher than that of MoS_2_ NSs aqueous dispersion, indicating the photothermal property of MoS_2_ NSs was not significantly influenced by surface modification. What's more, MoS_2_ NSs and MP NSs showed similar temperature evolution during heating (laser-on) and cooling (laser-off) processes (Figures S4A,B). After laser irradiation at 0.43 W/cm^2^ for 30 min, UV-Vis-NIR absorption spectra of MoS_2_ NSs and MP NSs showed no obvious change (Figures S4C,D), indicating good photothermal stability.

### Stability, Protein Loading Ability, and Biocompatibility of MPPI NSs

**Figure 3:**
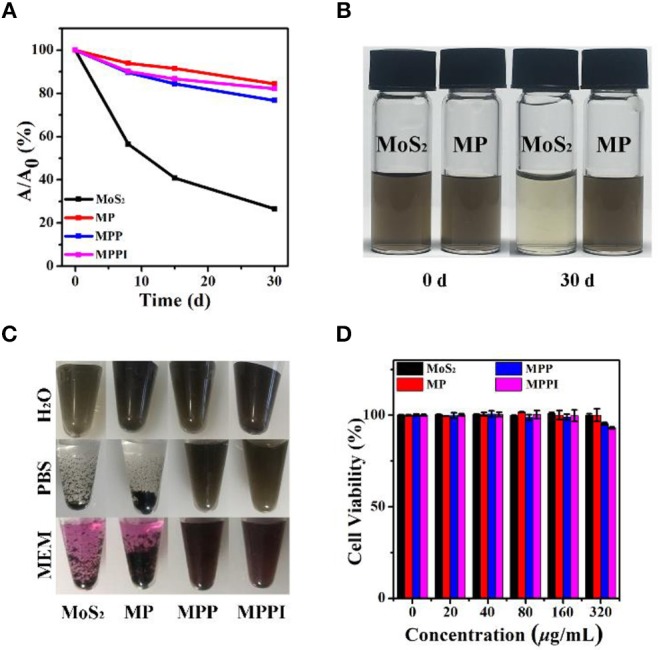
**(A)** The relative absorbance at 785 nm of MoS_2_ NSs, MP NSs, MPP NSs, and MPPI NSs aqueous dispersions (MoS_2_: 20 μg/mL) after stored in ambient environment at different times. **(B)** Photographs of MoS_2_ NSs and MP NSs aqueous dispersions (MoS_2_: 20 μg/mL) at 0 d and 30 d. **(C)** Photographs of MoS_2_ NSs, MP NSs, MPP NSs, and MPPI NSs (MoS_2_: 40 μg/mL) dispersed in H_2_O, PBS (phosphate buffered saline), and MEM (Minimum Essential Medium) for 1 d, respectively. **(D)** Cell viabilities of human cervical carcinoma (HeLa) cells after incubation with MoS_2_ NSs, MP NSs, MPP NSs, and MPPI NSs for 24 h.

The stability of MoS_2_ NSs, MP NSs, MPP NSs, and MPPI NSs was studied by comparing the absorbance of MoS_2_ at 785 nm at different times. As shown in Figure 3A, the absorbance of MoS_2_ NSs aqueous dispersion (20 μg/mL) at 785 nm decreases rapidly. The absorbance of MoS_2_ NSs at 30 d is <30% of that at the beginning, due to the oxidation of MoS_2_ NSs. By contrast, the absorbance of MP NSs, MPP NSs, and MPPI NSs aqueous dispersions (MoS_2_: 20 μg/mL) at 30 d stays more than 80% compared with that at 0 d, indicating greatly improved stability. As shown in Figure 3B, the color of MoS_2_ NSs aqueous dispersion is much lighter at 30 d than that at 0 d, while the MP NSs aqueous dispersion at 30 d is almost the same as that at 0 d, suggesting the same trend as the change of absorbance. MoS_2_ NSs is stable in pure water, but usually aggregate in salt-containing buffer (Zhang et al., 2017). As shown in Figure 3C, MoS_2_ NSs and MP NSs (MoS_2_: 40 μg/mL) aggregated completely in phosphate buffered saline (PBS) and Minimum Essential Medium (MEM) after 1 d. In contrast, MPP NSs and MPPI NSs remained well dispersed in H_2_O, PBS, and MEM (MoS_2_: 40 μg/mL) without any obvious precipitates even after 14 d (Figure S5), and their hydrodynamic sizes almost remained the same (Figure S6), demonstrating great colloidal stability of MPP NSs and MPPI NSs.

The conjugation efficiency of protein to MP NSs was investigated by using bovine serum albumin (BSA) as an example. Determined by Bradford protein assay (Compton and Jones, 1985), 1 mg of MoS_2_ NSs can load 0.012 mg of BSA, while MP NSs (containing 1 mg MoS_2_) can load 0.434 mg of BSA, suggesting a 36-fold increase.

Biocompatibility is an essential factor for the biomedical application of nanomaterials. As illustrated in Figure 3D, HeLa cells remained almost 100% viable after incubation with MoS_2_ NSs and MP NSs, and more than 90% viable with MPP NSs and MPPI NSs even at the concentration of up to 320 μg/mL, demonstrating good biocompatibility of MPPI NSs.

### Targeting Ability of MPPI NSs to *S. aureus* Biofilms

**Figure 4:**
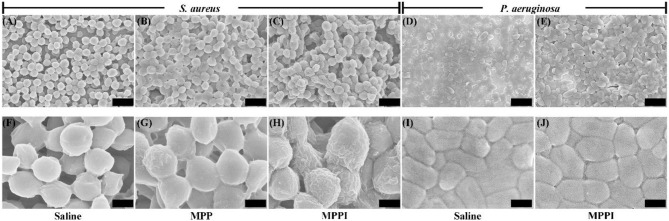
Representative scanning electron microscopy (SEM) images of *S. aureus* biofilms after 6 h incubation with saline **(A,F)**, MPP NSs (**B,G**, MoS_2_: 160 μg/mL), and MPPI NSs (**C,H**, MoS_2_: 160 μg/mL) and *P. aeruginosa* biofilms with saline **(D,I)** and MPPI NSs (**E,J**, MoS_2_: 160 μg/mL). The scale bars represent 2 μm in **(A–E)** and 500 nm in **(F–J)**.

Prior to the photothermal therapy, the targeting ability of MPPI NSs to *S. aureus* biofilms was studied. As shown in Figure S7, the planktonic *S. aureus* cells have smooth, clear, and spherical morphology, while the surface of bacterial cells in *S. aureus* biofilms are less clear due to the enclosing of EPS (Figures 4A,F; Asahi et al., 2015). As indicated in Figures 4B,G, crumpled accumulation of nanosheets can be observed on the surface of a few *S. aureus* cells in biofilms after 6 h incubation with MPP NSs (MoS_2_: 160 μg/mL) due to the non-specific absorption. In contrast, most *S. aureus* cells in biofilms were covered by crumpled nanosheets after incubation with MPPI NSs (MoS_2_: 160 μg/mL) for 6 h (Figures 4C,H), suggesting effectively binding of MPPI NSs to *S. aureus*. To confirm the targeting ability of MPPI NSs to *S. aureus* biofilms, *Pseudomonas aeruginosa* (*P. aeruginosa*) biofilms was also incubated with MPPI NSs (MoS_2_: 160 μg/mL) for 6 h. SEM images show that no obvious change can be observed between the *P. aeruginosa* biofilms treated with saline (Figures 4D,I) and that with MPPI NSs (Figures 4E,J). The specific accumulation of MPPI NSs in *S. aureus* biofilms was also analyzed by energy dispersive X-ray spectroscopy (EDS). As illustrated in Figure S8, the atomic percentages of Mo among all elements (C, N, O, S, P, and Mo) in *S. aureus* biofilms cultured with MPP NSs and *P. aeruginosa* biofilms cultured with MPPI NSs are very low (0.08~0.1%) even at high concentration (MoS_2_: 160 μg/mL), suggesting that limited amount of nanosheets bind to biofilms through non-specific adsorption. In contrast, the atomic percentage of Mo in *S. aureus* biofilms cultured with MPPI NSs (MoS_2_: 160 μg/mL) is 0.48%, which is about 5-fold of that with MPP NSs. These results demonstrate the excellent *S. aureus* biofilm-targeting ability of MPPI NSs.

As shown in Figure S9, the human prostatic stromal myofibroblast cell line (WPMY-1 cells) stayed almost 100% viable after PTT of MPPI NSs (0.58 W/cm^2^ for 10 min), even when the concentration of MPPI NSs reached up to 160 μg/mL. The WPMY-1 cells after PTT of MPPI NSs were also co-stained by calcein-AM and propidium iodide (PI). Live cells were stained green by calcein, while dead cells were stained red by PI. Figure S10 shows barely red fluorescence, and almost all WPMY-1 cells show green fluorescence, indicating that the targeted-PTT of MPPI NSs has little side effects to normal mammalian cells.

### *In vitro* Targeted PTT of *S. aureus* Biofilms

**Figure 5:**
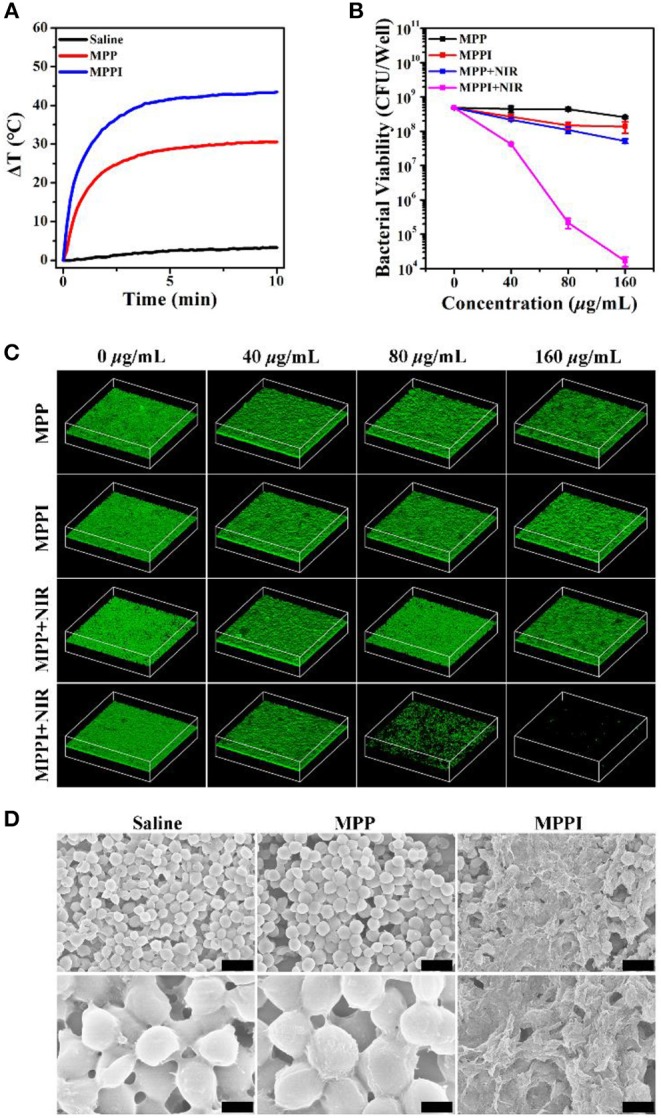
*In vitro* targeted PTT of *S. aureus* biofilms. **(A)** Temperature changing curves of *S. aureus* biofilms under 785 nm laser irradiation (0.58 W/cm^2^, 10 min) after 6 h incubation with saline, MPP NSs, and MPPI NSs (MoS_2_: 160 μg/mL), respectively. **(B)** Bacterial viability and **(C)** Three dimensional (3D) confocal laser scanning microscopy (CLSM) images (630 × 630 μm) of *S. aureus* biofilms with or without NIR laser irradiation after incubation with different concentrations of MPP NSs or MPPI NSs. **(D)** Representative SEM images of *S. aureus* biofilms after PTT of MPP NSs or MPPI NSs (MoS_2_: 160 μg/mL). The scale bars in upper row and lower row represent 2 μm and 500 nm, respectively.

Prior to the *in vivo* treatment study of *S. aureus* biofilm-related infection, the *in vitro* PTT efficacy of *S. aureus* biofilms by MPPI NSs was evaluated. As shown in Figure 5A, the temperature of *S. aureus* biofilms rose by 2°C under 785 nm laser irradiation (0.58 W/cm^2^, 10 min). The *S. aureus* biofilms with MPP NSs and MPPI NSs incubation and NIR laser irradiation show temperature increase of 30 and 43°C, respectively, suggesting more MPPI NSs accumulate in *S. aureus* biofilms than MPP NSs due to the specific binding mediated by antibody.

As shown in Figure 5B, the colony forming units (CFU) of *S. aureus* biofilms after the treatments of MPP NSs and MPPI NSs incubation (MoS_2_: 160 μg/mL) decreases by about 0.36 log (~57.08%) and 0.77 log (~77.07%), respectively. The CFU of *S. aureus* biofilms decreases by 0.96 log (~89.14%) after PTT of MPP NSs (MoS_2_: 160 μg/mL), while that decreases by 4.46 log (>99.99%) after PTT of MPPI NSs (MoS_2_: 160 μg/mL), showing excellent targeted PTT efficacy.

Three dimensional (3D) confocal laser scanning microscopy (CLSM) was used to directly observe live bacteria in biofilms that were stained green by calcein (Chen et al., 2016). As shown in Figure 5C, the intensity of green fluorescence shows limited reduction in *S. aureus* biofilms of control groups, including MPP, MPPI, and MPP + NIR groups, which indicates the MPP NSs, MPPI NSs, and non-targeted PTT of MPP NSs have neglectable antibiofilm efficacy. In contrast, after targeted PTT of MPPI NSs, the green fluorescence of *S. aureus* biofilms decreases along with the increase of the concentration of MPPI NSs, and nearly vanishes at the concentration of 160 μg/mL, indicating that almost all *S. aureus* in biofilms are killed. The significant difference of the green fluorescence between MPPI + NIR group and MPP + NIR group suggests that the antibiofilm efficacy of targeted PTT is much better than the non-targeted PTT, due to the limited range of photo-induced thermal effect. These results are consistent with the changing trend of CFU numbers after different treatments.

As shown in Figure 5D, the bacteria in preformed *S. aureus* biofilms have intact morphology after NIR laser irradiation (Saline) or PTT of MPP NSs (MPP). On the contrary, after PTT of MPPI NSs (MPPI), lysed bacterial morphology and debris can be observed, suggesting serious structural damage of *S. aurues* biofilms. The structure change of *S. aureus* biofilms after PTT of MPPI NSs was also revealed by crystal violet staining (Chen et al., 2016). As shown in Figure S11, the *S. aureus* biofilms after PTT of MPPI NSs become loosened, indicating that PTT of MPPI NSs can partially destroy the EPS-encased structure of biofilms. Hence, *S. aureus* biofilms can be inactivated by targeted photothermal ablation using MPPI NSs efficiently.

### *In vivo* PTT of *S. aureus* Focal Infection

**Figure 6:**
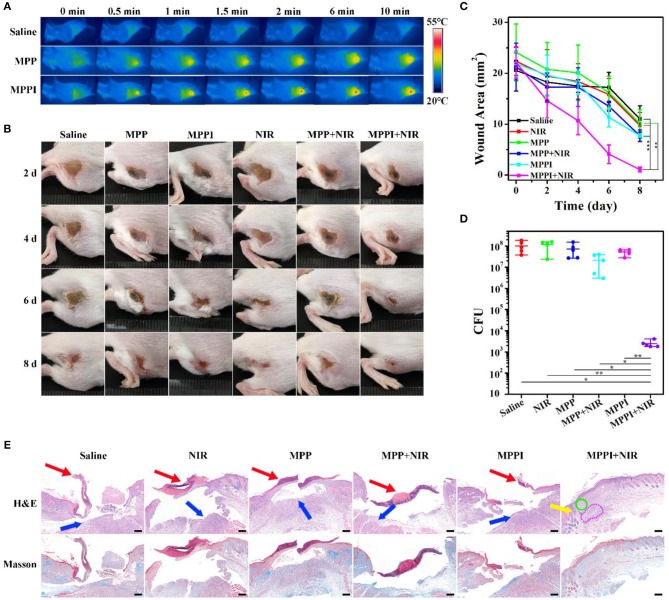
*In vivo* PTT of *S. aureus* focal infection. **(A)** IR thermal images of *S. aureus* infected tissues under 785 nm laser irradiation (0.58 W/cm^2^, 10 min) at 12 h after *in situ* injection (into the abscess) of saline (100 μL), MPP NSs (100 μL, MoS_2_: 40 μg/mL), and MPPI NSs (100 μL, MoS_2_: 40 μg/mL). **(B)** Photographs (2 cm × 2 cm) of the *S. aureus* infected tissues at different times after treatments. **(C)** The wound area of subcutaneous abscesses after various treatments. **(D)** Colony forming units (CFU) of the *S. aureus* infected tissues at 8th day after different treatments. **(E)** Microphotographs of hematoxylin and eosin (H&E) stained and Masson's trichrome stained slices of the *S. aureus* infected tissues at 8th day post-treatment. Red arrows and blue arrows indicate superficial ulcer in the epidermal layer and inflammatory cell infiltration in the dermal layer, respectively. Yellow arrow, green circle, and pink circle indicate hair follicle formation, fibroblast proliferation, and granulomatous inflammation in the dermal layer, respectively. Scale bar represents 200 μm. Data represent mean ± SD (*n* = 5), *p < 0.05, **p < 0.01, and ***p < 0.001. Statistical analysis by two-tailed unpaired *t*-test.

To construct *S. aureus* focal infection model, *S. aureus* suspensions were subcutaneously injected into the right flanks of Balb/c mice (Hu et al., 2017; Zhao et al., 2017). These mice with subcutaneous abscesses were randomly divided into six groups: Saline, NIR, MPP, MPP + NIR, MPPI, and MPPI + NIR. Among them, 100 μL of MPP NSs or MPPI NSs dispersed in saline were injected into the *S. aureus* focal infection sites with a dose of 4 μg (40 μg/mL), and saline was used as control. NIR laser irradiation (785 nm, 0.58 W/cm^2^, 10 min) was carried out at 12 h post-injection. As shown in Figure 6A and Figure S12, the temperature of *S. aureus* infected skins treated with saline rose from 33 to 34°C under NIR laser irradiation, while the temperatures of infected tissues with PTT of MPP NSs and MPPI NSs increased to ~43 and ~50°C, respectively. The higher temperature indicates that more MPPI NSs accumulate at the infection sites than MPP NSs.

The PTT efficacy of MPPI NSs was studied during the following 8 days after treatments. As shown in Figures 6B,C, the mice from MPPI + NIR group showed much earlier wound scarring and significantly faster healing than those from the other five control groups (Saline, MPP, MPPI, NIR, and MPP + NIR). Wound crust appeared at 4th day after PTT of MPPI NSs, and fell off at 6th day. After 8 d, the *S. aureus* infected tissues almost recovered with a small scar left. As to the five control groups, there were no crust formed until 6 d after treatments. At 8th day, the crust detached, but ulceration still remained, showing much slower healing. As shown in Figure 6C, the average area of infected tissues from MPPI + NIR group was reduced to ~1 mm^2^ at 8th day post-treatment, while those from other five control groups were still larger than 7 mm^2^.

The number of viable *S. aureus* cells from the infected tissues were obtained by standard plate counting method at 8th day post-treatment. As shown in Figure 6D and Figure S13, the CFU of the infected tissues from three groups (NIR, MPP, and MPPI), shows <0.25 log reduction compared to the Saline group, suggesting that neither MPP NSs or MPPI NSs incubation nor NIR laser irradiation can kill the bacteria effectively. The number of bacteria in infected tissues after PTT of MPPI NSs is reduced by more than 4 log (>99.99%), which is significantly higher than the group treated with PTT of MPP NSs (0.57 log, ~48.43%).

Hematoxylin and eosin (H&E) staining and Masson's trichrome staining were used to evaluate the healing status of *S. aureus* infected tissues. As shown in Figure 6E, the infected tissues from the five control groups (Saline, NIR, MPP, MPP + NIR, and MPPI) exhibited severe inflammation on the 8th day post-treatment, including superficial ulcer in the epidermal layer (indicated by red arrows), massive inflammatory cells infiltration in the dermal layer (indicated by blue arrows, and also shown in Figure S14), and collagen layer (stained blue) disappearing. On the contrary, the infected skin of mice after PTT of MPPI NSs showed intact epidermal layer with dense collagen fibers, and proliferation of fibroblast (indicated by green circle) as well as hair follicles (indicated by yellow arrow) in the dermal layer. Although there was still a little bit of granulomatous inflammation (indicated by pink circle), the *S. aureus* focal infection almost healed at 8th day after the targeted PTT of MPPI NSs. These results demonstrate the superiority of targeted PTT for the treatment of *S. aureus* focal infection over non-targeted PTT.

### *In vivo* Toxicity

**Figure 7:**
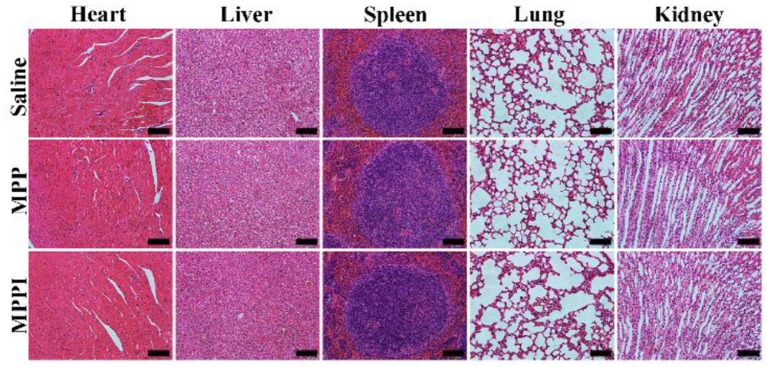
Microphotographs of H&E stained slices of major organs of Balb/c mice at 30th day after i.v. injection of saline, MPP NSs, and MPPI NSs. The MPP NSs and MPPI NSs contained 500 μg/mL of MoS_2_, and the injection volume was 200 μL. The scale bar represents 200 μm.

As illustrated in Figure 7, the major organs (heart, liver, spleen, lung, and kidney) of mice with i.v. injection of MPP NSs or MPPI NSs have similar morphology as the healthy tissues (Saline) without noticeable organ damage or inflammatory lesion. Although more studies of long-term toxicity are still required, MPPI NSs show no obvious *in vivo* toxicity at the dose we used.

## Discussion

PTT is a promising alternative solution of antibiotics for the treatment of bacterial biofilm-related diseases. Targeting functionalization has great potential to improve the therapeutic efficiency and reduce the side effects. In this direction, the modification of targeting moieties to photothermal agents would be a key factor. *S. aureus* is one of the leading causes of biofilm-related infections (Otto, 2013; Tong et al., 2015). Protein A is a surface protein in the cell wall of *S. aureus*. Anti-protein A IgG has proven effective to target *S. aureus*, and used here as the targeting moiety (Meeker et al., 2016, 2018).

MoS_2_ NSs is an excellent PTT agent with good biocompatibility, high extinction coefficient and photothermal conversion efficiency in the NIR region (Robinson et al., 2011; Chou et al., 2013; Li et al., 2017). MoS_2_ NSs can be functionalized by various biomolecules, such as PEG-SH, proteins, DNA, and so on, through coordination interaction and physical absorption (Li et al., 2017). These methods are easy to carry out, and the intrinsic properties of MoS_2_ NSs would not be affected (Li et al., 2017). Nevertheless, because of these weak non-covalent conjugation, biomolecules tend to desorb from the surface of MoS_2_ NSs, which would cause the invalidation of the functionalization and destabilization, especially in physiological environment. Therefore, a facile method for valid and stable functionalization is in demand.

It is realizable for dopamine to polymerize on the surface of MoS_2_ NSs, and the *in situ* polymerization of dopamine would deposit a layer of PDA on the surface of MoS_2_ NSs, yielding MP NSs (Yuwen et al., 2018). Moreover, PDA has plenty of catechol groups that can facilitate the covalent linkage with amine and thiol groups via Michael addition and/or Schiff base reactions (Liu et al., 2014). Thus, we use PDA as interface of MoS_2_ NSs to conjugate PEG-SH and anti-protein A IgG covalently to form MPPI NSs, as illustrated in Scheme 1. The surface modification with PDA, PEG-SH, and antibody is moderate without causing any damage to the morphology of MoS_2_ NSs (Figures 1A–D), but increases the thickness (Figures 1E–H). The PDA coating not only enhances the stability of MoS_2_ NSs against oxidation (Figures 3A,B), but also greatly improves the protein-loading efficiency (36-fold increase). As a result, the MPPI NSs have better colloidal stability compared with MoS_2_ NSs (Figure 3C, Figures S5, S6), good biocompatibility (Figure 3D), and excellent photothermal property (Figure 2D) and stability (Figure S4).

As shown in Figure 4 and Figure S8, much more MPPI NSs accumulate in *S. aureus* biofilms than MPP NSs without IgG functionalization (5-fold increase), resulting higher temperature under NIR laser irradiation (Figure 5A). Although the amount of MPPI NSs and MPP NSs at the infection sites of mice was not measured quantitatively, the higher temperature of *S. aureus*-infected skins treated with MPPI NSs than that with MPP NSs indicates that more MPPI NSs accumulate at the infection sites than MPP NSs (Figure 6A and Figure S12). These results demonstrate that anti-protein A IgG provides MoS_2_ NSs with specific binding ability to the *S. aureus* cells in both biofilms and infection sites through the antibody-antigen interaction with high binding affinity. What's more, the antibody-antigen interaction can shorten the distance between *S. aureus* cells and photothermal agents, which could enhance the ablation effect of local photothermal hyperpyrexia (Peng et al., 2018; Zhang et al., 2018). Combining the efficient accumulation and reduced distance of MPPI NSs to *S. aureus*, the targeted PTT of MPPI NSs achieved an excellent inactivation efficiency of more than 99.99% both *in vitro* and *in vivo*, and eventually accelerated the healing of *S. aureus* focal infection (Figures 5, 6). Meanwhile, normal mammalian cells are barely affected by the PTT of MPPI NSs (Figures S9, S10), indicating little side effects of the targeted-PTT. In one word, a *S. aureus*-targeting PTT agent has been proposed with enhanced therapeutic efficiency and low side effects, suggesting the necessity of targeting functionalization when designing PTT agents.

## Conclusions

In this study, MPPI NSs were prepared for targeted PTT of *S. aureus* focal infection. With PDA coating, MoS_2_ NSs can be functionalized covalently with PEG-SH and IgG. As a result, MPPI NSs possess excellent colloidal stability, conjugation efficiency, photothermal property, biocompatibility, and especially *S. aureus*-targeting ability. MPPI NSs can accumulate in *S. aureus* biofilms effectively and specifically with an amount of almost 5 times more than MPP NSs without IgG. The numbers of *S. aureus* in biofilms and infected tissues were reduced by more than 4 log (>99.99%) by targeted PTT of MPPI NSs, which was significantly higher than that of MPP NSs without targeting ability (<90% *in vitro* and <50% *in vivo*). Meanwhile, the PTT of MPPI NSs show no harm to normal mammalian cells, demonstrating the low side effects of targeted PTT. Targeted PTT of MPPI NSs also shows much faster healing process for the treatment of *S. aureus* focal infection *in vivo* than non-targeted PTT. With no obvious toxicity observed both *in vitro* and *in vivo*, these results demonstrate that the MPPI NSs have great potential as a targeted PTT agent for the treatment of *S. aureus* infection.

## Data Availability

All datasets generated for this study are included in the manuscript/Supplementary Files.

## Ethics Statement

The animal study was reviewed and approved by the Animal Ethics Committee of Nanjing University.

## Author Contributions

LWa and LY conceived and supervised the study. YZ and WX designed and carried out the experiments, and analyzed the results. SG and JS helped with the *in vivo* experiments. SR conducted the SEM and EDS measurements. YZ wrote the manuscript. All authors participated in revising the paper.

### Conflict of Interest Statement

The authors declare that the research was conducted in the absence of any commercial or financial relationships that could be construed as a potential conflict of interest.
