# Supplementary material for: Antibody-Functionalized MoS2 Nanosheets for Targeted Photothermal Therapy of Staphylococcus aureus Focal Infection
Source: Front Bioeng Biotechnol. 2019 Sep 10;7:218. doi: 10.3389/fbioe.2019.00218 (PMC6746923; doi:10.3389/fbioe.2019.00218)
Supplement: Supplementary file 1 [file Table_1.pdf]

Supplementary Material

1 Characterization

Transmission electron microscopy (TEM, Hitachi HT-7700, 120 kV) and atomic force microscopy (AFM, BRUKER Dimension Icon with ScanAsyst) were used to characterize the morphology of the nanosheets. Ultraviolet-visible-near infrared (UV-Vis-NIR) absorption spectra and Fourier-transform infrared (FT-IR) spectra were recorded on a Shimadzu UV-3600 spectrophotometer and a PerkinElmer FT-IR spectrometer, respectively. X-ray photoelectron spectroscopy (XPS) spectra were obtained from PHI 5000 VersaProbe with an A1 Kα X-ray source. The hydrodynamic sizes and Zeta potentials of the nanosheets were analyzed with a ZetaPALS Potential Analyzer (Brookhaven). Scanning electron microscopy (SEM, Hitachi S-4800) and energy dispersive X-ray spectroscopy (EDS, Horiba EX-250X-Max) were used for imaging and elemental analysis of bacterial biofilms. A Fortic 225 infrared imaging camera was used for thermal imaging and temperature recording, and the temperature evolution during PTT was analyzed by the AnalyzIR software.

2 Supplementary Figures

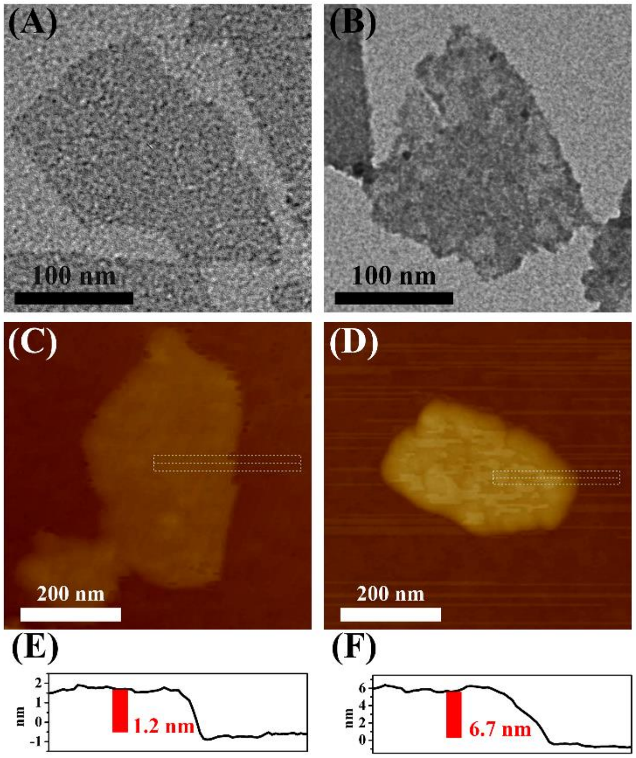

Figure S1. Transmission electron microscopy (TEM) images of (A) MoS_2 NSs and (B) MP NSs; atomic force microscopy (AFM) images and height profiles of (C, E) MoS_2 NSs and (D, F) MP NSs.

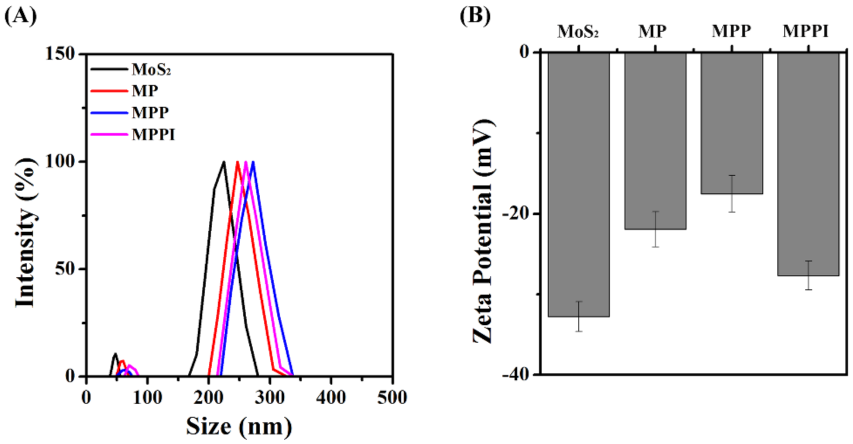

Figure S2. (A) Hydrodynamic sizes determined by dynamic light scattering (DLS) and (B) Zeta potentials of MoS_2 NSs, MP NSs, MPP NSs, and MPPI NSs.

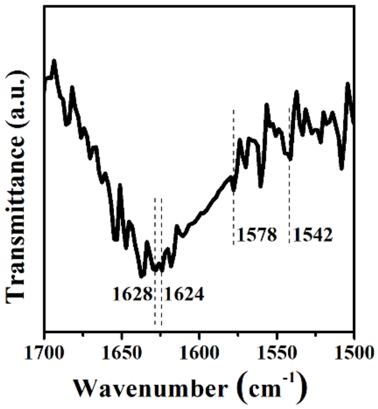

Figure S3. Locally enlarged Fourier transform infrared spectroscopy (FT-IR) spectrum of MPPI NSs.

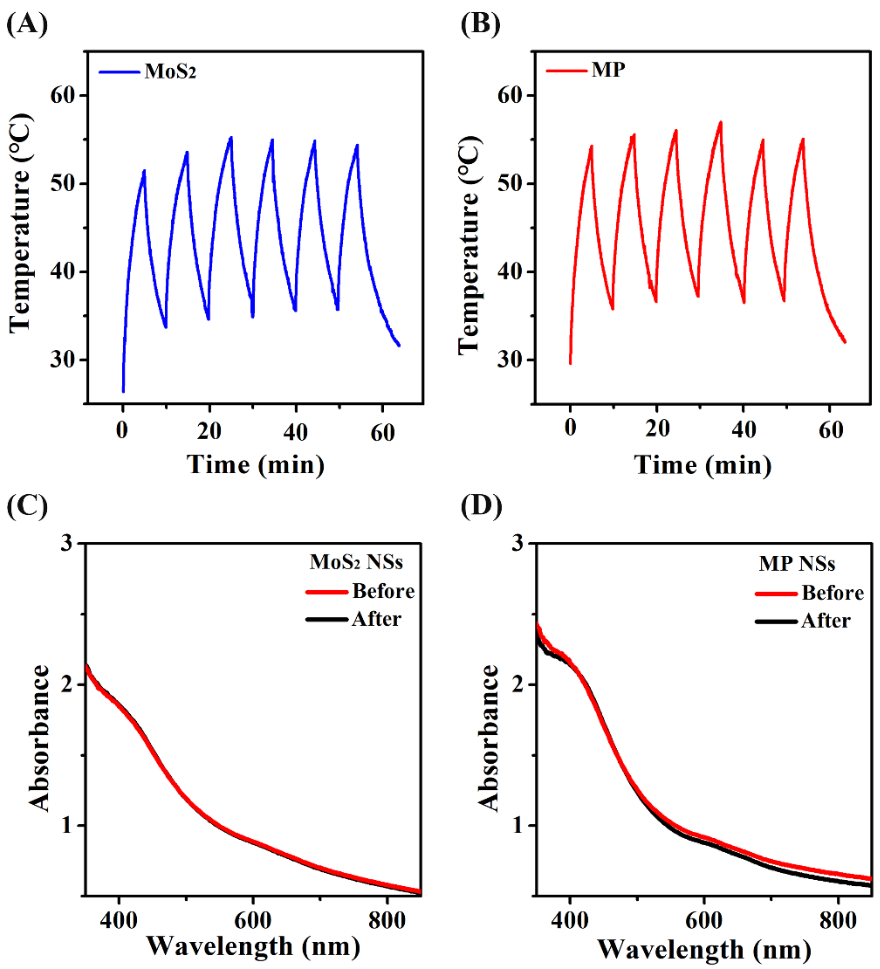

Figure S4. Temperature evolution curves of (A) MoS_2 NSs and (B) MP NSs aqueous dispersion (MoS_2: 30 μg/mL) during heating (laser on) and cooling (laser off); UV-Vis-NIR absorption spectra of (C) MoS_2 NSs and (D) MP NSs before and after laser irradiation for 30 min. The NIR laser irradiation was performed with a 785 nm laser at the power density of 0.43 W/cm^2.

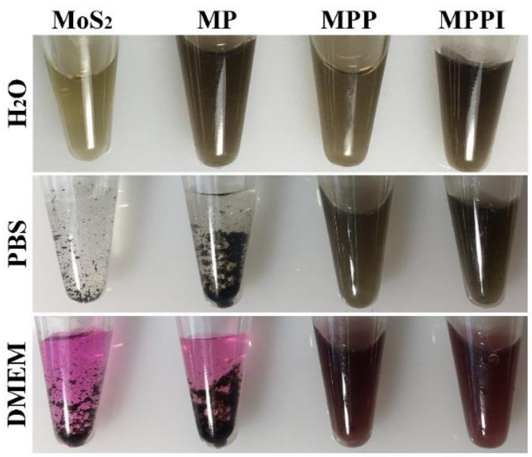

Figure S5. Photographs of MoS_2 NSs, MP NSs, MPP NSs, and MPPI NSs dispersed in H_2O, PBS, and MEM for 14 d. All the aqueous dispersions contained 40 μg/mL of MoS_2.

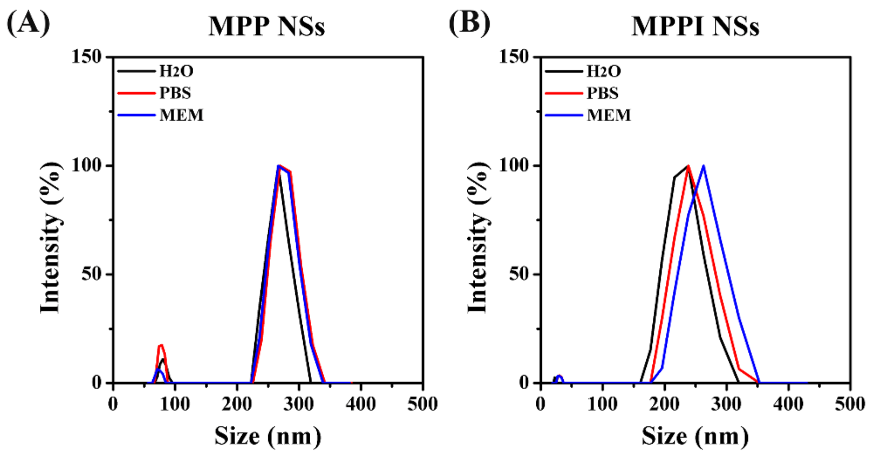

Figure S6. Hydrodynamic sizes determined by DLS of (A) MPP NSs and (B) MPPI NSs.

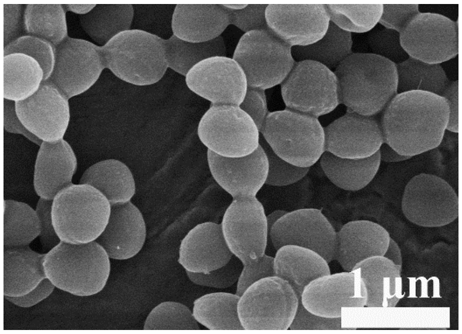

Figure S7. SEM image of planktonic *S. aureus*.

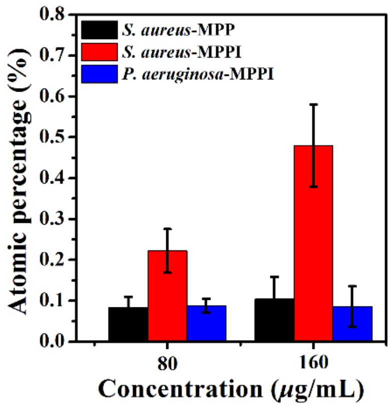

Figure S8. Atomic percentages of Mo element among all elements (Mo, C, N, O, P, and S) in *S. aureus* and *P. aeruginosa* biofilms detected by EDS after incubation with MPP NSs and MPPI NSs of different concentrations for 6 h, respectively.

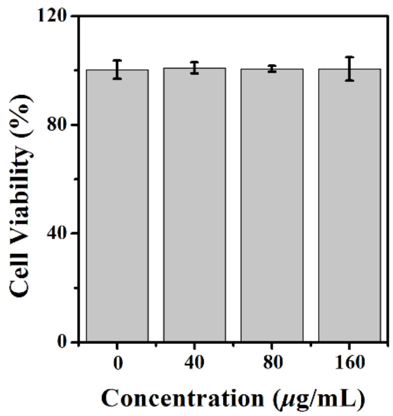

Figure S9. Cell viability of WPMY-1 cells after PTT with different concentrations of MPPI NSs dispersions. The concentration of MoS_2 in MPPI NSs are 40, 80, and 160 μg/mL, respectively. The NIR laser irradiation was performed by using a 785 nm laser at 0.58 W/cm^2 for 10 min. Cell viability was detected with a Lactate Dehydrogenase (LDH)-Cytotoxicity Colorimetric Assay Kit.

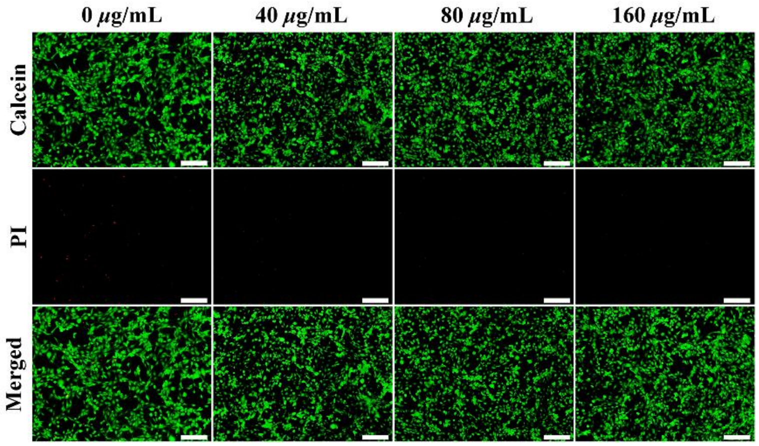

Figure S10. Fluorescence microscopy images of WPMY-1 cells co-stained by calcein-AM and PI after PTT. The cells were incubated with MPPI NSs for 6 h, washed, and irradiated with 785 nm laser at the power density of 0.58 W/cm^2 for 10 min. Live cells are stained green, while dead cells are stained red. The scale bar represents 20 μm.

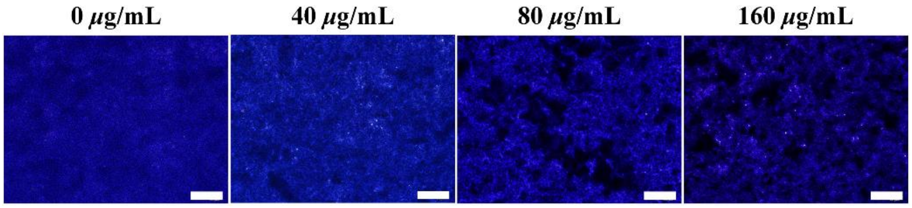

Figure S11. *S. aureus* biofilms stained by crystal violet after PTT of MPPI NSs. The biofilms grown in 96-well plates were incubated with different concentrations of MPPI NSs for 6 h, and then irradiated by 785 nm laser at the power density of 0.58 W/cm^2 for 10 min. The scale bar represents 100 μm.

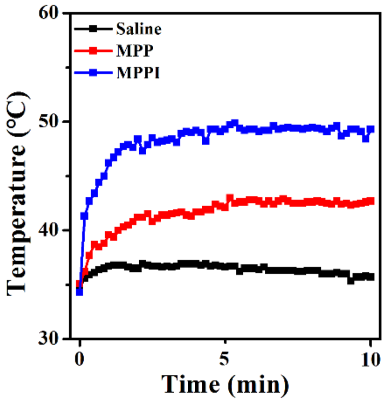

Figure S12. Temperature evolution curves of *S. aureus* infected skins during NIR laser irradiation (785 nm, 0.58 W/cm^2, 10 min) after in situ injection of saline, MPP NSs, and MPPI NSs. The MPP NSs and MPPI NSs saline dispersions contained 40 μg/mL of MoS_2, and the injection volume was 100 μL.

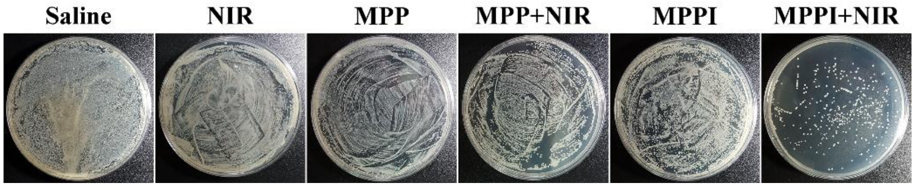

Figure S13. Photographs of bacterial culturing plates from the *S. aureus* infected tissues at 8th day post-treatment.

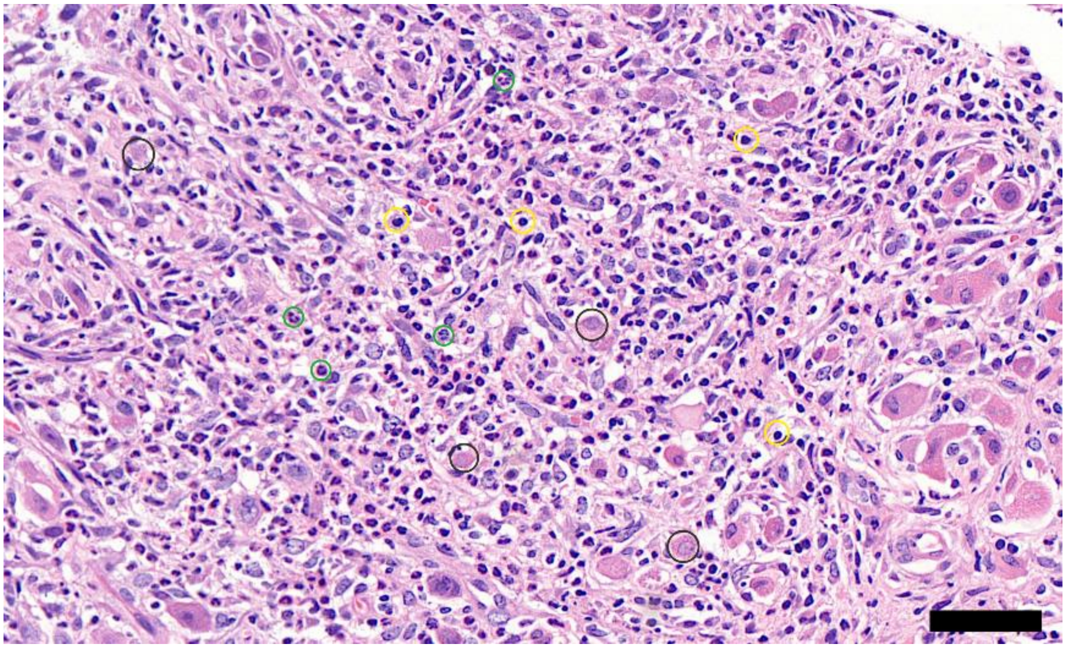

Figure S14. Inflammatory cells infiltration in the dermal layer of the *S. aureus* infected tissues stained by hematoxylin and eosin (H&E). Neutrophile granulocyte, lymphocyte, and multinuclear giant cells are indicated by green, yellow, and black circles, respectively. The scale bar represents 100 μm.
